# Abundance of the Fanconi anaemia core complex is regulated by the RuvBL1 and RuvBL2 AAA+ ATPases

**DOI:** 10.1093/nar/gku1230

**Published:** 2014-11-26

**Authors:** Eeson Rajendra, Juan I. Garaycoechea, Ketan J. Patel, Lori A. Passmore

**Affiliations:** 1 MRC Laboratory of Molecular Biology, Francis Crick Avenue, Cambridge, CB2 0QH, UK; 2 Department of Medicine, Level 5, Addenbrooke's Hospital, University of Cambridge, Cambridge, CB2 0QQ, UK

## Abstract

Fanconi anaemia (FA) is a genome instability disease caused by defects in the FA DNA repair pathway that senses and repairs damage caused by DNA interstrand crosslinks. At least 8 of the 16 genes found mutated in FA encode proteins that assemble into the FA core complex, a multisubunit monoubiquitin E3 ligase. Here, we show that the RuvBL1 and RuvBL2 AAA+ ATPases co-purify with FA core complex isolated under stringent but native conditions from a vertebrate cell line. Depletion of the RuvBL1-RuvBL2 complex in human cells causes hallmark features of FA including DNA crosslinker sensitivity, chromosomal instability and defective FA pathway activation. Genetic knockout of RuvBL1 in a murine model is embryonic lethal while conditional inactivation in the haematopoietic stem cell pool confers profound aplastic anaemia. Together these findings reveal a function for RuvBL1-RuvBL2 in DNA repair through a physical and functional association with the FA core complex. Surprisingly, depletion of RuvBL1-RuvBL2 leads to co-depletion of the FA core complex in human cells. This suggests that a potential mechanism for the role of RuvBL1-RuvBL2 in maintaining genome integrity is through controlling the cellular abundance of FA core complex.

## INTRODUCTION

Fanconi anaemia (FA) is a genetic disease that is characterized by a complex phenotype including bone marrow failure, developmental defects and a strong predisposition to cancer (1,2). At the cellular level, there is a defect in the response to endogenous aldehydes and chemotherapeutic agents that introduce DNA interstrand crosslinks (ICLs), such as mitomycin C (MMC) (3–5). When challenged with ICLs, cells from FA patients display profound genomic instability due to defective DNA repair.

There are currently 16 known FANC genes that have been described to be inactivated in FA patients (FANCA to FANCQ), and 5 FA-associated gene products (FAAPs) (1). Eleven of these proteins (FANC-A, -B, -C, -E, -F, -G, -L and -M plus FAAP100, FAAP24 and FAAP20) associate to form the FA core complex, a multi-subunit E3 ubiquitin ligase that is activated upon DNA damage (6). The roles of many of the FA core complex subunits are unknown since they have no significant homology to proteins of known function. FANCM is a helicase and may facilitate recruitment to chromatin. The RING-finger containing FANCL subunit, which functions in the context of the intact FA core complex, mediates the specific monoubiquitination of FANCD2 and FANCI on chromatin (7–11). This modification is a critical activating step in the pathway. Ubiquitinated FANCD2-FANCI likely serve as a platform for the recruitment of additional proteins that orchestrate DNA repair (2). These include nucleases (FAN1 and the scaffold protein FANCP/SLX4 that recruits three other nucleases FANCQ/XPF, SLX1 and MUS81), a helicase (FANCJ) and components of homologous recombination (HR) repair machinery (FANCD1/BRCA2, FANCN/PALB2, FANCO/RAD51C) (12).

Stimulation of this FA DNA repair pathway occurs downstream of the global DNA damage response (DDR) which involves activation of ATR and ATM—two kinases of the phosphatidylinositol 3-kinase-related kinase (PIKK) family. Through a coordinated cascade of phosphorylation, either directly, or through their effector kinases CHK1 and CHK2, respectively, a wide variety of substrates are modified to coordinate cell cycle checkpoint activation and DNA repair mechanisms (13). ATR has a key role in the response to replication stress during S-phase, activating the FA pathway and HR (14–16). The mechanism of this activation step is unclear; ATR has been shown to phosphorylate the FA core complex and the substrates FANCD2 and, crucially, FANCI (17). This latter event is essential for substrate monoubiquitination by the FA core complex.

The RuvBL1 and RuvBL2 proteins (also known as pontin/TIP49/TIP49A and reptin/TIP48/TIP49B, respectively) are highly conserved and are homologous to the bacterial RuvB DNA helicase (18,19). They encode adenosine triphosphatases (ATPases) associated with diverse cellular activities (AAA+) (20,21), which likely assemble into two hexameric rings to form a dodecamer (22,23). The majority of studies show that RuvBL1 and RuvBL2 are found together and function in complex with each other. Separable roles for the individual proteins have also been proposed but whether they actually function independently of each other has not been resolved (24–26). The RuvBL1-RuvBL2 complex is reported to play roles in chromatin remodelling and transcription (for review, see (25,26)). For example, RuvBL1-RuvBL2 associates with the TIP60 histone acetyltransferase complex (27) and the SWR1/SRCAP and INO80 chromatin remodelling complexes (28–30). RuvBL1-RuvBL2 also associates with the PIKK family of proteins that includes ATM, ATR, mTOR, DNA-PKcs, SMG-1 and TRRAP (31,32). In all cases, it appears that RuvBL1-RuvBL2 facilitates assembly and/or stability of the proteins. However, the detailed mechanism of how RuvBL1-RuvBL2 performs these roles is unclear.

Several lines of evidence support a role of RuvBL1-RuvBL2 in the DDR. First, studies have shown that RuvBL1-RuvBL2 is involved in the cellular response to certain types of damage, e.g. ultraviolet (33,34) and ionizing (35) radiation. Secondly, RuvBL1 and RuvBL2 are components of distinct complexes that serve roles in DNA repair, such as INO80 (36), TIP60 (37), SWR1/SRCAP (38) and YY1 (39–41). It is unknown if they function directly in repair processes or simply stabilize these larger assemblies allowing them to perform their other enzymatic roles in chromatin remodelling. Thirdly, several studies support a key role in facilitating HR as Rad51 foci formation is impaired upon RuvBL1-RuvBL2 depletion (41,42). At which step of DNA repair RuvBL1-RuvBL2 functions to impinge upon HR remains elusive.

We recently described the biochemical purification of intact native FA core complex from avian DT40 cells and reconstituted site-specific monoubiquitination of the FANCD2 substrate *in vitro* (43). In the present study, we identify RuvBL1 and RuvBL2 as *bona fide* interactors of the FA core complex. We show that depletion of the RuvBL1-RuvBL2 complex in human cells causes hallmark features of FA including DNA crosslinker sensitivity, and elevated spontaneous and crosslinker-induced chromosomal instability. At the molecular level, there is a specific defect in the monoubiquitination of FANCI, and a severe reduction in the abundance of the FA core complex at both mRNA and protein levels. Our work defines a direct role for the RuvBL1-RuvBL2 AAA+ ATPases in controlling the abundance of the FA core complex and therefore regulating DNA crosslink repair.

## MATERIALS AND METHODS

### Mammalian transfection

For small interfering RNA (siRNA) treatment, U2OS cells were reverse transfected with 25 nM siRNA (Dharmacon and Qiagen; Supplementary Table S1) using Dharmafect 1 reagent (Dharmacon) according to the manufacturer's protocol. Unless stated otherwise, cells were incubated for 48 h with indicated siRNAs. 1 μM MMC or vehicle control was added for a further 24 h. For western blotting and immunofluoresence, 2.5 × 10^5^ cells/well of a 6-well plate were transfected. For metaphase spreads and subcellular fractionation, 1.2 × 10^6^ cells were transfected in a 10 cm dish.

### Drug sensitivity assay

U2OS cells were reverse transfected with siRNA in 96-well plate format at a density of 5000 cells/well. After 24 h, MMC was added and cells were subsequently incubated for a further 72 h. Alternatively, after 48 h, cisplatin was added and cells were incubated for a further 48 h. CellTiter-Blue Cell Viability Assay reagents (Promega) were added, incubated at 37°C for 3 h, then analyzed using a Fusion Plate Reader at 560 nm excitation/590 nm emission. Each experiment was conducted in triplicate and repeated multiple times. Cell viability was normalized to the viability of cells after siRNA treatment but in the absence of MMC.

### Purification of the FA core complex

Purification and mass spectrometry of the FA core complex from DT40 cells in which the genomic copies of *FANCB* were deleted and a tandem affinity purification (TAP)-tagged version of *FANCB* (*FANCB-GS*) was used to complement the cells (ΔB/B-GS) have been described (43).

### Real-time quantitative polymerase chain reaction (PCR)

RNA was isolated from U2OS cells after siRNA treatment using the RNeasy mini kit (Qiagen). cDNA was synthesized using the QuantiTect Reverse Transcription Kit (Qiagen). Real-time quantitative PCR was performed using the QuantiTect SYBR Green PCR kit (Qiagen) on an ABI Real Time 7900HT PCR machine using the QuantiTect Primer Assays (Qiagen; Supplementary Table S2).

### Whole cell extract (WCE) preparation and western blotting

Cells were washed in phosphate buffered saline, harvested by trypsinization and collected by centrifugation. WCEs were prepared by lysis in ice-cold RIPA buffer (50 mM Tris-HCl, pH 7.4, 150 mM NaCl, 0.5% sodium deoxycholate, 0.1% sodium dodecyl sulphate (SDS) and 1% Igepal CA-630 (Sigma)) freshly supplemented with 1 mM dithiothreitol (DTT), 1 mM phenylmethanesulfonyl fluoride (PMSF), 2 mM Na_3_VO_4_, 25 mM NaF, 40 mM β-glycerophosphate, 1× Halt Phosphatase Inhibitor Cocktail (Pierce) and a Protease Inhibitor Cocktail (Roche). Lysates were cleared by centrifugation. Protein concentration was quantified using Bicinchoninic Assay (BCA) (Thermo Scientific). WCEs (30 μg per sample) were resolved by SDS- polyacrylamide gel electrophoresis (SDS-PAGE) on 4–12% Bis-Tris gels or 3–8% Tris-Acetate gels (Invitrogen) and transferred to polyvinylidene fluoride (PVDF) membranes (Millipore) before blotting with the appropriate antibodies (Supplementary Table S3).

### Mice

*Ruvbl1^+/–^* mice were obtained from the European Mouse Mutant Archive (*Ruvbl1^tm1a(EUCOMM)Wtsi^*; MGI code: 4432034, C57BL/6N). Targeting was confirmed by long-range PCR using the following oligonucleotides: LAR3 CACAACGGGTTCTTCTGTTAGTCC; Ruv1F2 GTCCATGTGACATTTCTGTGTATG; RAF5 CACACCTCCCCCTGAACCTGAAAC; and Ruv1R3 CAACCCTGAAAGAGATGGTAAATG. *Ruvbl1^+/–^* mice were first crossed with FLPeR mice (44) to generate *Ruvbl1^+^/Flox* mice. These were further crossed with Lmo2-CreERT2 mice and intercrossed to generate *Ruvbl1*Flox/Flox Lmo2-CreERT2^+^ mice and controls. Lmo2-CreERT2 mice carry a knock-in of CreERT2 into exon 4 of *Lmo2* and were a gift from Terence Rabbitts (L. Bao, O. Al-Asar, M. Metzler, J. Chambers, T.H. Rabbitts, in preparation). Genotyping was performed using the following oligonucletotides: Ruv1F1 TGGTGTCTGTGGTAGTTCTGCTTG; Ruv1R1 TGCGGAAGTTCTCCATCAGC; and En2A GCTTCACTGAGTCTCTGGCATCTC. Cre recombination was induced by injecting 8- to 12-week-old mice intraperitoneally with 1 mg of tamoxifen (dissolved in sunflower oil at 10 mg/ml), once a day on five consecutive days. Peripheral blood was collected in ethylenediaminetetraacetic acid (EDTA) microvette tubes (Starstedt) and analyzed on a VetABC analyser (Horiba). Histological analysis was performed on femurs that had been fixed in neutral buffered formalin for 24 h, paraffin embedded and stained with haematoxylin and eosin (H&E). All animals were maintained in specific pathogen-free conditions. All animal experiments undertaken in this study were done so with the approval of the UK Home Office.

### Statistical analysis

All statistical analyses were performed using GraphPad Prism version 5 for Mac (GraphPad Software).

## RESULTS

### RuvBL1 and RuvBL2 associate with a purified FA core complex

**Figure 1.**
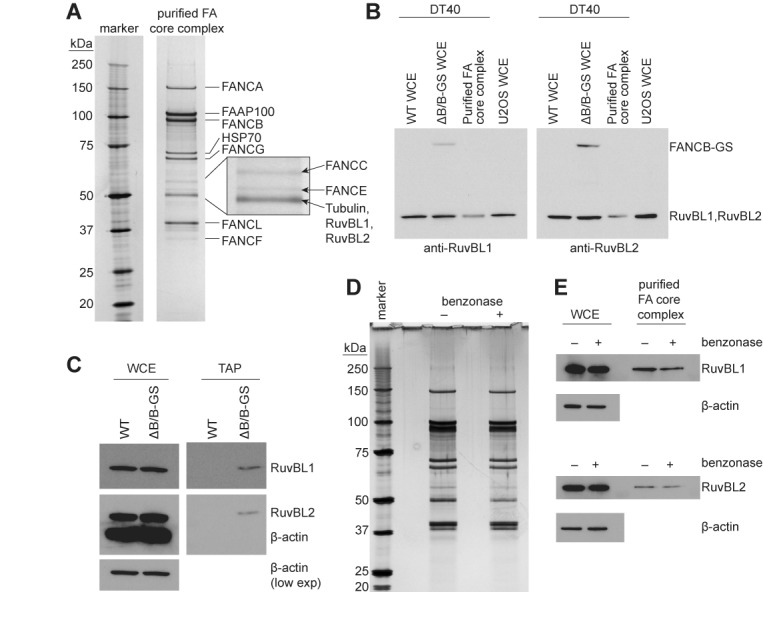
RuvBL1-RuvBL2 associates with purified chicken FA core complex. (**A**) FA core complex was purified from ΔB/B-GS DT40 chicken cells and analyzed by 4–12% SDS-PAGE followed by silver staining. Bands were excised from multiple independent purifications and analyzed by mass spectrometry for identification. The inset shows the distinct migration of FANCC, FANCE and Tubulin/RuvBL1/RuvBL2. (**B**) The presence of RuvBL1 and RuvBL2 was confirmed by western blotting of the purified FA core complex, and WCEs from DT40 (‘WT’ and ‘ΔB/B-GS’) and from human (U2OS) cells. The IgG-binding moiety of protein G in the TAP tag is detected with the antibody against RuvBL1 or RuvBL2. (**C**) RuvBL1 and RuvBL2 co-purify with the FA core complex but are not present in a mock purification. TAP was performed in parallel from wild-type (WT) and ΔB/B-GS DT40 cells. RuvBL1 and RuvBL2 were detected by western blotting and found only in the purified FA core complex. β-actin is shown as a loading control. (**D** and **E**) Association of RuvBL1-RuvBL2 with the FA core complex is not dependent on nucleic acids. TAP was performed in the presence or absence of benzonase during the lysis and IgG-agarose affinity steps to remove any nucleic acids. The purified complexes were separated by 4–12% SDS-PAGE and analyzed by silver-staining (**D**). Western blotting (**E**) shows the presence of RuvBL1 (top) and RuvBL2 (bottom) in these purified complexes. There are no significant changes in the FA core complex and RuvBL1 and RuvBL2 remain associated after benzonase treatment.

We recently reported the purification of intact and biochemically active FA core complex from avian DT40 cells under native conditions using a TAP strategy via a tagged FANCB subunit (43). The presence of known core complex subunits in the purification was confirmed by mass spectrometry and western blotting. Because we used a stringent, multi-step purification method with gentle elution, all co-purifying proteins must have a robust interaction, directly or indirectly, with the core complex. Interestingly, a band migrating slightly faster than FANCE was clearly visible on the gels (Figure 1A). This band would co-migrate with the immunoglobulin G (IgG) heavy chain, and therefore may not have been visible in previous immunopurification approaches to isolate the FA core complex. Mass spectrometry revealed that this 50 kDa band contained tubulin (16 unique peptides covering 52.6% of the sequence), RuvBL1 (10 unique peptides covering 32.2%) and RuvBL2 (10 unique peptides covering 27.8%; Supplementary Figure S1).

We confirmed that purified FA core complex interacts with RuvBL1 and RuvBL2 using western blotting with specific antibodies (Figure 1B and Supplementary Figure S2). Importantly, neither FA core complex subunits nor RuvBL1-RuvBL2, were present in a mock purification (Figure 1C). Further, we were unable to find any differences in the purification after nuclease treatment, demonstrating that the association is not dependent on nucleic acid (Figure 1D and E). Thus, RuvBL1 and RuvBL2 co-purify with the FA core complex, indicating a robust association in cycling cells.

### RuvBL1-RuvBL2 depletion confers cellular sensitivity to DNA crosslinking agents

**Figure 2.**
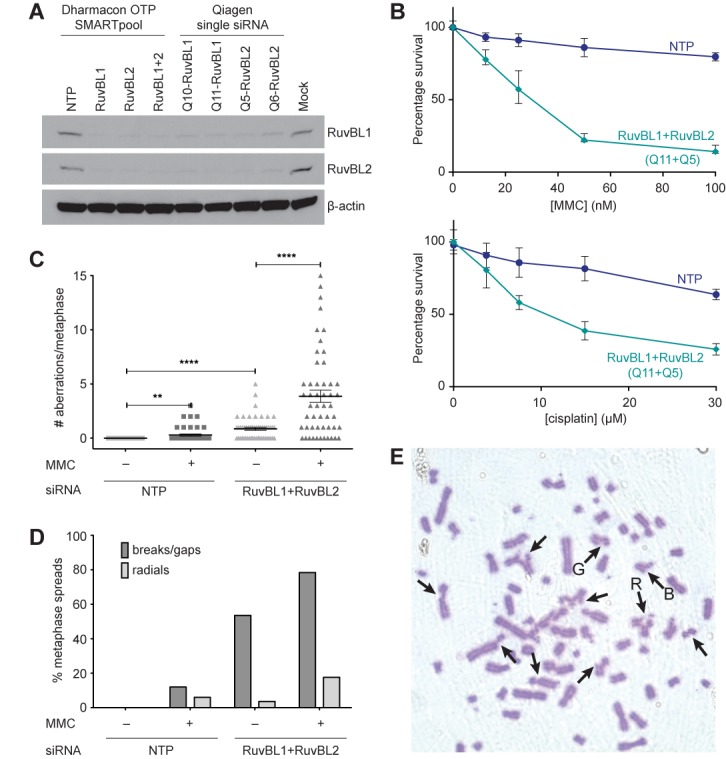
siRNA-mediated depletion of RuvBL1-RuvBL2 in human cells confers sensitivity to DNA crosslinking agents and chromosomal instability. (**A**) Knockdown of the RuvBL1-RuvBL2 complex in human U2OS cells was performed with either a pool of siRNA targeting the coding region of the gene (Dharmacon OTP SMARTpool) or independent single siRNAs targeting the 3'-UTR (Qiagen single siRNA). Blotting of WCEs with the indicated antibodies after 48 h of siRNA treatment showed co-depletion of the non-targeted RuvBL protein in the heteromeric complex. NTP is a negative control non-targeting pool siRNA and Mock is a mock-transfected sample. (**B**) Cell viability was monitored after knockdown of the RuvBL1-RuvBL2 complex with single siRNAs targeting both proteins and treatment with MMC (top panel) or cisplatin (bottom panel). Each point represents the mean of triplicate samples from two independent experiments and the error bars are the SD. (**C**) Scatter plot showing the presence of aberrant metaphases in cells treated with a non-targeting siRNA (NTP) or siRNAs against RuvBL1 and RuvBL2 in the presence or absence of MMC treatment. At least 50 metaphases were scored per sample. The central line represents the mean and the error bars are the SEM. **P-value < 0.01; ****P-value < 0.0001 (unpaired *t*-test). Chromosomal aberrations are quantified in the bar chart (D) showing the percentages of metaphases harbouring chromosome breaks/gaps (dark grey) and/or radial structures (light grey). (**E**) Representative image of a metaphase spread from U2OS cells treated with siRNAs against both RuvBL1 and RuvBL2 after MMC treatment. Chromosome gaps (‘G’), chromosome breaks (‘B’) and radial structures (‘R’) are indicated by arrows.

RuvBL1 and RuvBL2 are AAA+ ATPases that interact with each other in a dodecameric structure of two hexameric rings (22,23,45). In human U2OS cells, siRNA depletion of one subunit results in co-depletion of the other indicating that they form an obligate complex (Figure 2A). This is in agreement with reports that demonstrate coordinate regulation of RuvBL1 and RuvBL2 at the protein level (46).

The RuvBL1-RuvBL2 complex has been implicated in DNA repair through its roles in activating PIKKs and facilitating chromatin accessibility (31,47). Since RuvBL1-RuvBL2 physically interacts with the FA core complex, we wanted to determine whether it plays a direct role in ICL repair, which is largely mediated through the FA pathway in higher eukaryotic cells. Thus, we depleted RuvBL1 and/or RuvBL2 in U2OS cells by multiple independent siRNAs and examined their ability to repair ICLs. Depletion of either RuvBL1 or RuvBL2 results in severe cellular sensitivity to the DNA crosslinkers MMC and cisplatin (Figure 2B and Supplementary Figure S3A). We found an increased frequency of chromosomal aberrations (including radial structures, chromosome gaps and chromosome breaks) upon RuvBL1-RuvBL2 depletion, both after treatment with MMC, and spontaneously, in untreated cells (Figure 2C–E). Importantly, we could rescue the MMC sensitivity caused by RuvBL1 knockdown using stable cell lines expressing siRNA-insensitive FLAG-RuvBL1 (Supplementary Figure S3B and C).

Sensitivity to DNA crosslinking agents and an increased frequency of aberrant chromosome structures are characteristic phenotypes of cells with a defective FA DNA repair pathway. Thus, our results indicate that the RuvBL1-RuvBL2 complex plays an important role in the response to ICLs and in the maintenance of genome stability, reminiscent of a role in the FA pathway.

### Activation and abundance of the FA core complex is reduced after RuvBL1-RuvBL2 depletion

**Figure 3.**
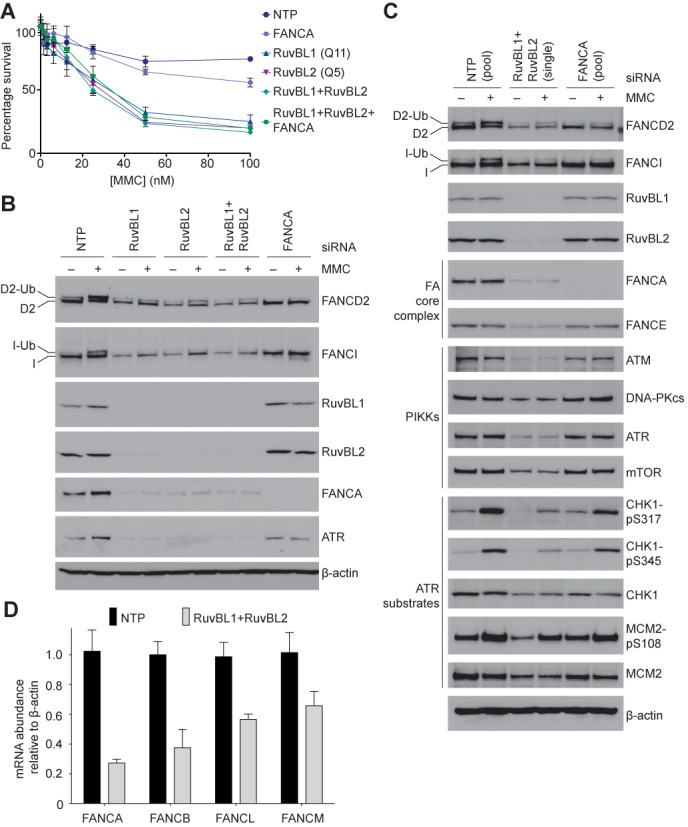
RuvBL1-RuvBL2 depletion impairs the activity and abundance of the FA core complex. (**A**) Cell viability was monitored in response to treatment with MMC after knockdown of RuvBL1, RuvBL2 and/or FANCA by siRNA targeting. Each point represents the mean of triplicate samples from two independent experiments and the error bars are the SD. (**B** and **C**) U2OS cells were treated with siRNAs targeting RuvBL1, RuvBL2, both RuvBL1 and RuvBL2 or FANCA, and exposed to MMC or left untreated. WCEs were western blotted with the indicated antibodies. Monoubiquitinated forms of FANCD2 and FANCI are indicated. (**B**) After RuvBL1-RuvBL2 depletion, FANCI ubiquitination was severely compromised and the abundance of ATR and FANCA were reduced. (**C**) In addition to reduced abundance of PIKK family members and impaired phosphorylation of ATR substrates, RuvBL1-RuvBL2 depletion conferred reduced levels of FA core complex subunits. (**D**) mRNA levels were analyzed by real-time quantitative PCR 72 h after transfection with RuvBL1 and RuvBL2 siRNAs (light grey) or a non-targeting siRNA pool (NTP, black). Cellular levels of indicated mRNAs were normalized to β-actin mRNA and are expressed relative to mRNA levels in untransfected cells. Bars indicate the mean and SEM based on data analyzed in duplicate from four independent experiments.

If the RuvBL1-RuvBL2 complex plays an important role in the FA pathway, additional depletion of a FA core complex protein should not further enhance sensitivity to ICLs. Indeed, we find that the MMC sensitivity of RuvBL1-RuvBL2-depleted cells is not enhanced further by simultaneous depletion of FANCA (Figure 3A and Supplementary Figure S4A). Depletion of the RuvBL1-RuvBL2 complex by siRNA was in itself cytotoxic, in agreement with several reports (48–56). Over the 96-h duration of the assay, RuvBL1-RuvBL2-depleted cells were more sensitive to MMC than FANCA-depleted cells. These results support a role for RuvBL1-RuvBL2 in the FA pathway, as well as much broader essential functions in cell viability beyond ICL repair.

The hallmark of FA pathway activation is the monoubiquitination of FANCD2 and FANCI; this is impaired by depletion of the FA core complex subunit FANCA (Figure 3B). Monoubiquitination provides a platform for the recruitment of nucleases essential for the incision of ICLs and thus triggers DNA repair. We found a modest defect in DNA damage-induced monoubiquitination of FANCD2 and a severe reduction in FANCI monoubiquitination upon depletion of RuvBL1-RuvBL2 (Figure 3B and Supplementary Figure S4B). This indicates that FA signalling is severely compromised. FANCI monoubiquitination can be rescued by siRNA-resistant FLAG-RuvBL1, but not by catalytically inactive FLAG-RuvBL1 D302N (Supplementary Figure S4C).

In agreement with a previously reported role in stabilizing PIKKs (31), we found a reduction in the levels of ATM, ATR, DNA-PKcs and mTOR, after RuvBL1-RuvBL2 depletion (Figure 3C). Unexpectedly, cells treated with RuvBL1-RuvBL2 siRNA also had drastically reduced levels of FANCA (Figure 3B and Supplementary Figure S4B). To determine whether loss of FANCA represents a general effect on the FA core complex, we examined the levels of another subunit, FANCE. FANCE is also depleted upon siRNA knockdown of RuvBL1-RuvBL2 but not after knockdown of FANCA (Figure 3C). FANCA and FANCE belong to distinct functional modules of the FA core complex (43,57,58). Thus, these results suggest that association with RuvBL1-RuvBL2 impacts the stability and/or assembly of the intact FA core complex. Moreover, loss of FA core complex provides a possible mechanism for the reduction in FANCI and FANCD2 monoubiquitination and cellular sensitivity to MMC after RuvBL1-RuvBL2 depletion.

FANCD2, FANCI and the FA core complex all localize to chromatin and form nuclear foci (10,11,59). The RuvBL1-RuvBL2 complex is proposed to regulate chromatin accessibility. Therefore, we reasoned that RuvBL1-RuvBL2 might function by facilitating loading (or unloading) of FA components onto DNA, and defects in this process may result in loss of the proteins through degradation. Using both cellular fractionation and immunofluorescence, we were unable to detect a significant effect of RuvBL1-RuvBL2 depletion on FANCD2, FANCI or FA core complex localization (Supplementary Figures S5 and S6). We conclude that the role of RuvBL1-RuvBL2 in the FA pathway is not likely mediated through changes in protein localization.

RuvBL1-RuvBL2 directly regulates the cellular abundance of all six PIKK family members at both the protein and mRNA levels *in vivo* (31,32). We performed real-time quantitative PCR to examine the effect of RuvBL1-RuvBL2 depletion on the mRNA levels of FA core complex subunits. Analogous to effects observed on PIKKs, after RuvBL1-RuvBL2 knockdown the mRNA levels of FA core complex subunits are reduced by 40–70% (Figure 3D). Flow cytometric analysis of RuvBL1-RuvBL2 depleted cells showed no evidence of a general proliferative block suggesting that the reduction in FA core complex mRNAs was unlikely to be cell cycle-mediated (Supplementary Figure S7). In addition to a protein chaperone-like activity, a transcriptional regulatory role has been attributed to RuvBL1-RuvBL2. For example, it associates with the TIP60 histone acetyltransferase and INO80 chromatin remodelling complexes (25–27,29). Neither TIP60 nor INO80 depletion has a significant effect on FA core complex abundance (Supplementary Figure S8). Together, these results show that RuvBL1-RuvBL2 proteins associate with the FA core complex and affect its abundance at both the protein and mRNA levels.

### Combined defects in FA and ATR signalling has a similar impact on ICL sensitivity as RuvBL1-RuvBL2 depletion

The PIKK family member ATR is a central player in the orchestration of the DDR (14,15). For example, FANCD2, FANCI and FA core complex subunits are ATR substrates (16,17,60–64). Since RuvBL1-RuvBL2 associates with both ATR and the FA core complex, we speculated that it might provide a direct link between sensing and repairing damaged DNA. Moreover, the effect of RuvBL1-RuvBL2 depletion on the FA pathway could, at least in part, be mediated through ATR.

**Figure 4.**
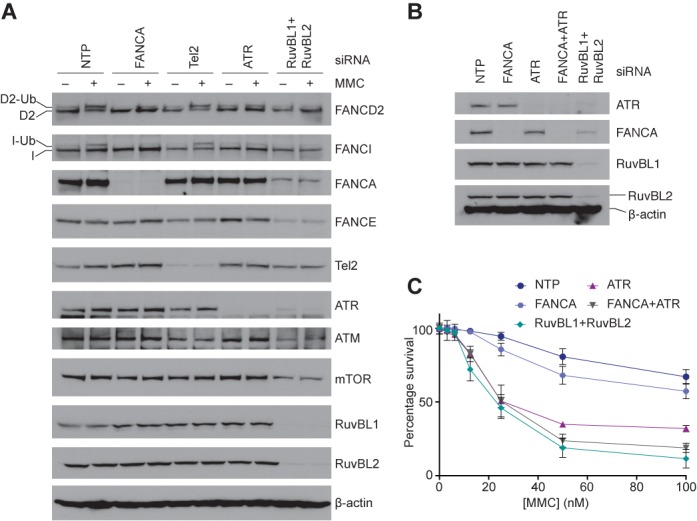
RuvBL1-RuvBL2 regulates both ATR and FA signalling pathways. (**A**) U2OS cells were treated with siRNA against FANCA, Tel2, ATR or RuvBL1 and RuvBL2 and exposed to MMC or left untreated. WCEs were western blotted with the indicated antibodies. Only RuvBL1-RuvBL2 depletion simultaneously impaired FANCI ubiquitination and reduced abundance of the FA core complex and PIKK family members. (**B**) Knockdown of ATR and the FA core complex component FANCA were performed with the indicated siRNAs, both in isolation and in combination, and WCEs were analyzed by western blotting with the indicated antibodies. For comparison, depletion of RuvBL1-RuvBL2 also reduces the cellular abundance of both ATR and FANCA. (**C**) Cell viability was monitored after depletion of RuvBL1 and RuvBL2, ATR and/or FANCA, followed by treatment with MMC. Each point represents the mean of triplicate samples from two independent experiments and the error bars are the SD.

We analyzed whether siRNA knockdown of ATR causes loss of FA core complex, similar to RuvBL1-RuvBL2 knockdown. After ATR depletion, there is a reduction of DNA damage-induced FANCD2-FANCI monoubiquitination (Figure 4A) (16,17). However, ATR depletion does not alter the abundance of the FA core complex (Figure 4A). Interestingly, upon RuvBL1-RuvBL2 depletion we observe a reduction in the levels of PIKKs (ATR, ATM, DNA-PKcs and mTOR) but key ATR substrates, such as the effector kinase CHK1, and MCM2 (65–67) are still phosphorylated (Figure 3C). Thus, ATR signalling is reduced but not completely abolished, in agreement with published data (31).

The stability/assembly of PIKKs is influenced by the interaction of RuvBL1-RuvBL2 with Tel2/hCLK2 and Hsp90 (68–72). Neither knockdown of Tel2 by siRNA, nor inhibition of Hsp90 by 17-N-allylamino-17-demethoxygeldanamycin (17-AAG) alters the global abundance of the FA core complex (Figure 4A and Supplementary Figures S8A and S9). Therefore, loss of the FA core complex is specific to RuvBL1-RuvBL2 depletion, is not a consequence of reduced ATR signalling, and is not directly mediated by Tel2 or Hsp90.

To further understand the relationship between ATR and the FA DNA repair pathway, which are both suppressed after RuvBL1-RuvBL2 depletion, we examined cellular sensitivity to MMC after knockdown of each of these proteins by siRNA: depletion of either FANCA or ATR results in sensitivity to MMC and this is more severe when they are co-depleted (Figure 4B and C). Interestingly, the combined loss of both FANCA and ATR results in a similar sensitivity to MMC as loss of RuvBL1-RuvBL2.

Taken together, our results show that RuvBL1-RuvBL2 plays a role in determining the abundance of the FA core complex. RuvBL1-RuvBL2 physically interacts with both FA core complex and ATR, and its effect on FA and ATR signalling is essential for the response to DNA crosslinking agents.

### Targeted inactivation of murine RuvBL1 in the haematopoietic stem cell (HSC) compartment confers bone marrow failure

**Figure 5.**
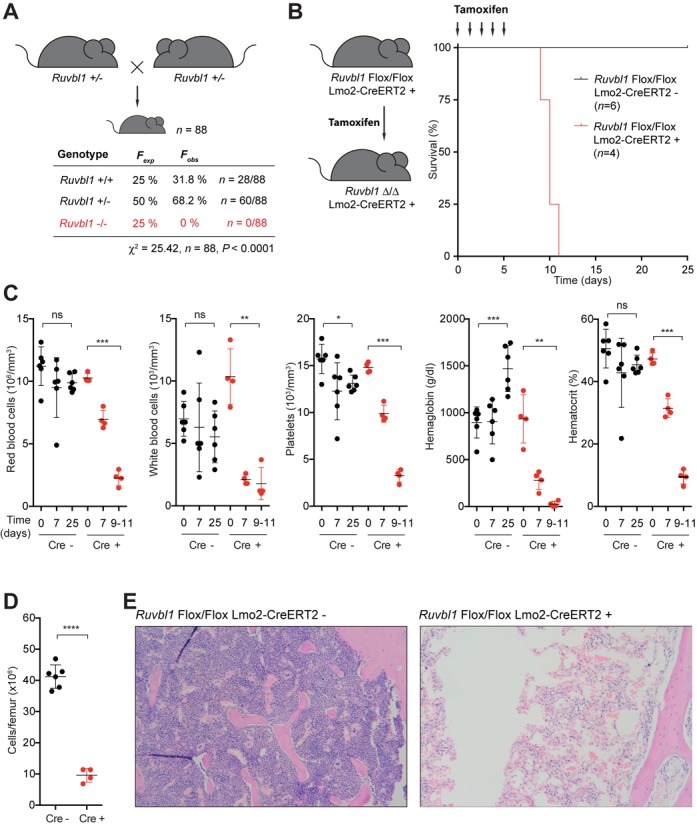
RuvBL1 is essential for mouse development and HSC maintenance. (**A**) *Ruvbl1*^+/-^ mice were mated to obtain *Ruvbl1*^-/-^ pups. *F*_exp_ and *F*_obs_ are expected and observed Mendelian frequencies, respectively, and *n*, the number of pups genotyped at three weeks after birth. No *Ruvbl1*^-/-^ pups were born (red). (**B**) Mice carrying a conditional disruption of the *Ruvbl1* locus were crossed with mice carrying the Lmo2-CreERT2 transgene, which drives the expression of the CreERT2 recombinase in HSCs (see Supplementary Figure S10). Treatment of *Ruvbl1* Flox/Flox Lmo2-CreERT2^+^ mice with tamoxifen is predicted to disrupt the *Ruvbl1* locus in the HSC compartment (*Ruvbl1* Δ/Δ Lmo2-CreERT2^+^). Between 9 and 11 days after tamoxifen treatment, these mice presented with severe aplastic anemia. (**C**) Analysis of the blood constituents revealed progressive anemia after tamoxifen treatment in *Ruvbl1* Flox/Flox Lmo2-CreERT2^+^ mice (red) but not controls (*Ruvbl1* Flox/Flox Lmo2-CreERT2^-^, black). (**D**) Quantification of the number of nucleated cells per femur and (**E**) bone marrow histology stained with H&E (×50) revealed profoundly hypocellular bone marrow in treated *Ruvbl1* Flox/Flox Lmo2-CreERT2^+^ mice but not controls. Error bars represent SEM. *, **, *** and **** indicate *P*-values <0.05, <0.01, <0.001 and <0.0001, respectively; ns, not significant.

To gain insight into the physiological importance of RuvBL1-RuvBL2 function we attempted to generate a RuvBL1 knockout mouse. In agreement with a recent report, homozygous knockout is embryonic lethal (Figure 5A and Supplementary Figure S10 (73)), corroborating findings in yeast (18,19) and *Drosophila* (74). Mouse knockouts of many FA proteins (but not FANCL) are viable (75–77), supporting additional roles for RuvBL1-RuvBL2 in essential cellular functions beyond FA repair.

FA can present with diverse and complex symptoms including bone marrow failure. To assess the specific role of RuvBL1 in HSC maintenance, we conditionally inactivated RuvBL1 in the HSC compartment (Figure 5B). Between 9 and 11 days, these mice presented with severe aplastic anaemia. Examination of blood components revealed a significant depletion of red blood cells, white blood cells, platelets, haemoglobin and haematocrit (Figure 5C) and was accompanied by concomitant loss of bone marrow (Figure 5D and E). Although FA pathway-deficient mice have quantifiable HSC defects, these do not lead to spontaneous bone marrow failure (78). Therefore, our findings support a role for RuvBL1 in the maintenance of HSCs that extends beyond its role in the FA pathway.

## DISCUSSION

In this study, we show that the RuvBL1-RuvBL2 AAA+ ATPases physically and functionally associate with the FA core complex. First, RuvBL1 and RuvBL2 co-purify with native FA core complex throughout a stringent multi-step purification. As the RuvBL1-RuvBL2 complex is a dodecamer, this association is likely substoichiometric. Secondly, depletion of RuvBL1-RuvBL2 in human cells confers cellular sensitivity to DNA crosslinking agents, and increases spontaneous and MMC-induced chromosomal instability. This establishes a role for RuvBL1-RuvBL2 in ICL repair. Thirdly, upon RuvBL1-RuvBL2 depletion, the abundance of the FA core complex is reduced and this impairs FANCD2-FANCI monoubiquitination and, consequently, DNA repair.

**Figure 6.**
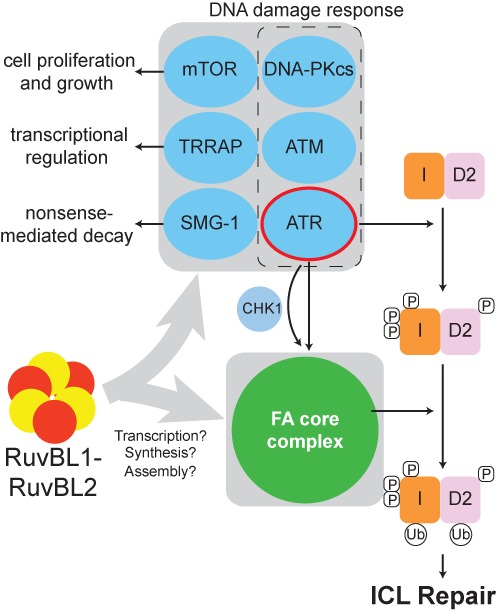
Model for the roles of the RuvBL1-RuvBL2 complex in regulating the FA DNA repair pathway. The RuvBL1-RuvBL2 complex interacts with both PIKKs and the FA core complex to regulate their abundance (grey boxes). Interaction with the PIKKs is required for robust activation of the global DDR through regulation of ATM and ATR. RuvBL1-RuvBL2 binds to and controls the abundance of the FA core complex (independent of DNA damage), thereby mediating the ability of the FA core complex to monoubiquitinate the FANCI-FANCD2 heterodimer and to activate the FA DNA repair response to ICLs.

The RuvBL1-RuvBL2 complex also regulates the DDR through control of the abundance of PIKK family members including ATR, ATM and DNA-PKcs. ATR in particular is recognized as the upstream damage-sensing kinase that activates the FA pathway (16,17,60). However, ATR does not regulate the abundance of the FA core complex—this is a function unique to RuvBL1-RuvBL2 (Figure 4A). A combined effect on the FA and ATR signalling pathways likely accounts for RuvBL1-RuvBL2's role in ICL repair (Figure 4C). This finding highlights the importance of combining checkpoint enforcement and repair for a robust DDR (Figure 6).

The physical and functional association of RuvBL1-RuvBL2 with the FA core complex strongly parallels its relationship with other multi-protein complexes. In all cases, RuvBL1-RuvBL2 physically interacts with a target protein, controls both mRNA and protein abundance of the target, and appears to influence assembly of the target into complexes. For example, RuvBL1-RuvBL2 is required for the assembly of SMG-1-containing mRNA surveillance complexes in nonsense-mediated mRNA decay (31). Similarly, it is required for assembly of the active ribonucleoprotein telomerase holoenzyme (46) and lack of RuvBL1-RuvBL2 affects assembly of INO80, resulting in loss of the Arp5 subunit and an inactive complex (79). Thus, RuvBL1-RuvBL2 may have a general role in mediating the assembly and abundance of macromolecular complexes. Although RuvBL1-RuvBL2 is proposed to interact with chaperoning machinery (80), we have been unable to find evidence of Pih1D1 and RPAP3 (components of the R2TP co-chaperone complex) in the purified FA core complex. Either these interacting proteins are present at very low levels or they do not play a role in the FA pathway.

The substoichiometric interaction of RuvBL1-RuvBL2 with FA core complex in our purification could imply direct binding to other low abundance components, such as FANCM. FANCM, and its associated proteins FAAP-10, -16 and -24 have been implicated in chromatin targeting and DNA recognition (81). Many of the complexes affected by RuvBL1-RuvBL2 involve nucleic acids (25,26) and it may facilitate chromatin remodelling at sites of DNA damage, through its DNA helicase activity (19,23,82) or via its role in chromatin remodelling complexes. Thus, an interaction with RuvBL1-RuvBL2 could allow the FA core complex to bind chromatin, to access its substrate proteins FANCD2 and FANCI, or to be released from chromatin.

The relative contribution of the effects on both mRNA and protein levels warrants further investigation: a role in controlling mRNA levels has been proposed through direct transcriptional regulation (41,83,84) and/or functions in non-sense-mediated mRNA decay (32). Provocatively, a direct physical association with proteins yet regulation at the mRNA level could imply coordinated control of their translation or post-translational regulation (e.g. through co-translational assembly of complexes, chaperoning individual subunits or the maturation of an intact active complex).

Alternatively, the effect on the mRNA abundance of FA core complex proteins could be functionally distinct from its physical association. For example, a more general role in transcriptional activation could affect many genes, subsets of which encode proteins that bind RuvBL1-RuvBL2. Interestingly, the most well studied complexes that associate with RuvBL1-RuvBL2 are all strongly implicated in aspects of chromatin biology suggesting a true functional role in chromatin metabolism (26). Ultimately, further work will be necessary to disentangle transcriptional regulation, macromolecular chaperoning and enzymatic roles of RuvBL1-RuvBL2.

The role of RuvBL1-RuvBL2 in the FA DNA repair pathway and the fact that depletion of RuvBL1-RuvBL2 leads to hallmark features of FA including MMC sensitivity and chromosomal instability, raises the possibility that its mutation may play a role in chromosomal breakage syndromes, such as Seckel syndrome (characterized by defective ATR signalling (85)) or FA. Interestingly, misassembly of the FA core complex (e.g. upon depletion of a single subunit FAAP20 (86,87)) is known to cause degradation of other subunits in some cases. This could provide a possible explanation for the reduction in FA core complex abundance after RuvBL1-RuvBL2 depletion, and could play a role in disease. More generally, knockdown of RuvBL1-RuvBL2 promotes genome instability and, therefore, RuvBL1-RuvBL2 may act as a tumour suppressor. RuvBL1 and RuvBL2 knockout mice are embryonic lethal and conditional knockout of RuvBL1 in haematopoietic tissues leads to bone marrow failure (Figure 5 and (73)), a feature of FA. Intriguingly, phenotypic analyses of a heterozygous RuvBL2 mutant mouse generated through an ENU mutagenesis screen revealed a defect in T-cell development suggesting haploinsufficiency (88). Similarly, conditional inactivation of RuvBL1 in the T-cell compartment demonstrates an essential role in early T-cell development (89). Future studies will be needed to determine whether misregulation of RuvBL1-RuvBL2 directly contributes to human disease.

This study expands the repertoire of protein complexes that are regulated, at multiple levels, by RuvBL1-RuvBL2 and provides insight into how their specific function in ICL repair may be mediated (Figure 6). The exact mechanisms of their function remains poorly understood and their roles in so many diverse processes that are essential for cell viability make them a challenge to study. As regulators of the abundance of ATM, ATR and the FA core complex at both protein and mRNA levels, they could be fundamental regulators of the global DDR. A very recent study has demonstrated that depletion of RuvBL1 leads to the coordinate downregulation in expression of key genes involved in cell cycle control and the maintenance of genome stability (56). These included components of the FA core complex, FANCG and FANCE. Thus, RuvBL1-RuvBL2 could emerge as a master regulator coordinating cell cycle checkpoints and genome stability pathways at the levels of transcription, translation and/or chaperoning. Dissecting these distinct roles will be an important challenge for the future.

## SUPPLEMENTARY DATA

Supplementary Data are available at NAR Online.

SUPPLEMENTARY DATA
