# Supplementary material for: Abundance of the Fanconi anaemia core complex is regulated by the RuvBL1 and RuvBL2 AAA+ ATPases
Source: Nucleic Acids Res. 2014 Nov 26;42(22):13736–48. doi: 10.1093/nar/gku1230 (PMC4267650; doi:10.1093/nar/gku1230)
Supplement: SUPPLEMENTARY DATA [file supp_42_22_13736__index.html]

Abundance of the Fanconi anaemia core complex is regulated by the RuvBL1 and RuvBL2 AAA+ ATPases — Abundance of the Fanconi anaemia core complex is regulated by the RuvBL1 and RuvBL2 AAA+ ATPases — SUPPLEMENTARY DATA 

# Abundance of the Fanconi anaemia core complex is regulated by the RuvBL1 and RuvBL2 AAA+ ATPases

## SUPPLEMENTARY DATA

**Files in this Data Supplement:**

- SUPPLEMENTARY DATA
