# Supplementary material for: Abundance of the Fanconi anaemia core complex is regulated by the RuvBL1 and RuvBL2 AAA+ ATPases
Source: Nucleic Acids Res. 2014 Nov 26;42(22):13736–48. doi: 10.1093/nar/gku1230 (PMC4267650; doi:10.1093/nar/gku1230)
Supplement: SUPPLEMENTARY DATA [file supp_gku1230_nar-02508-d-2014-File008.pdf]

SUPPLEMENTARY DATA

Abundance of the Fanconi Anaemia core complex is regulated by the RuvBL1 and RuvBL2 AAA+ ATPases

Eeson Rajendra[1], Juan I. Garaycoechea[1], Ketan J. Patel[1,2] and Lori A. Passmore[1*]

[1] MRC Laboratory of Molecular Biology, Francis Crick Avenue, Cambridge, CB2 0QH, UK
[2] Department of Medicine, Level 5, Addenbrooke's Hospital, University of Cambridge, Cambridge CB2 0QQ, UK
* To whom correspondence should be addressed. Tel: +44 1223 267062; Fax: + 44 1223 268305; Email: passmore@mrc-lmb.cam.ac.uk

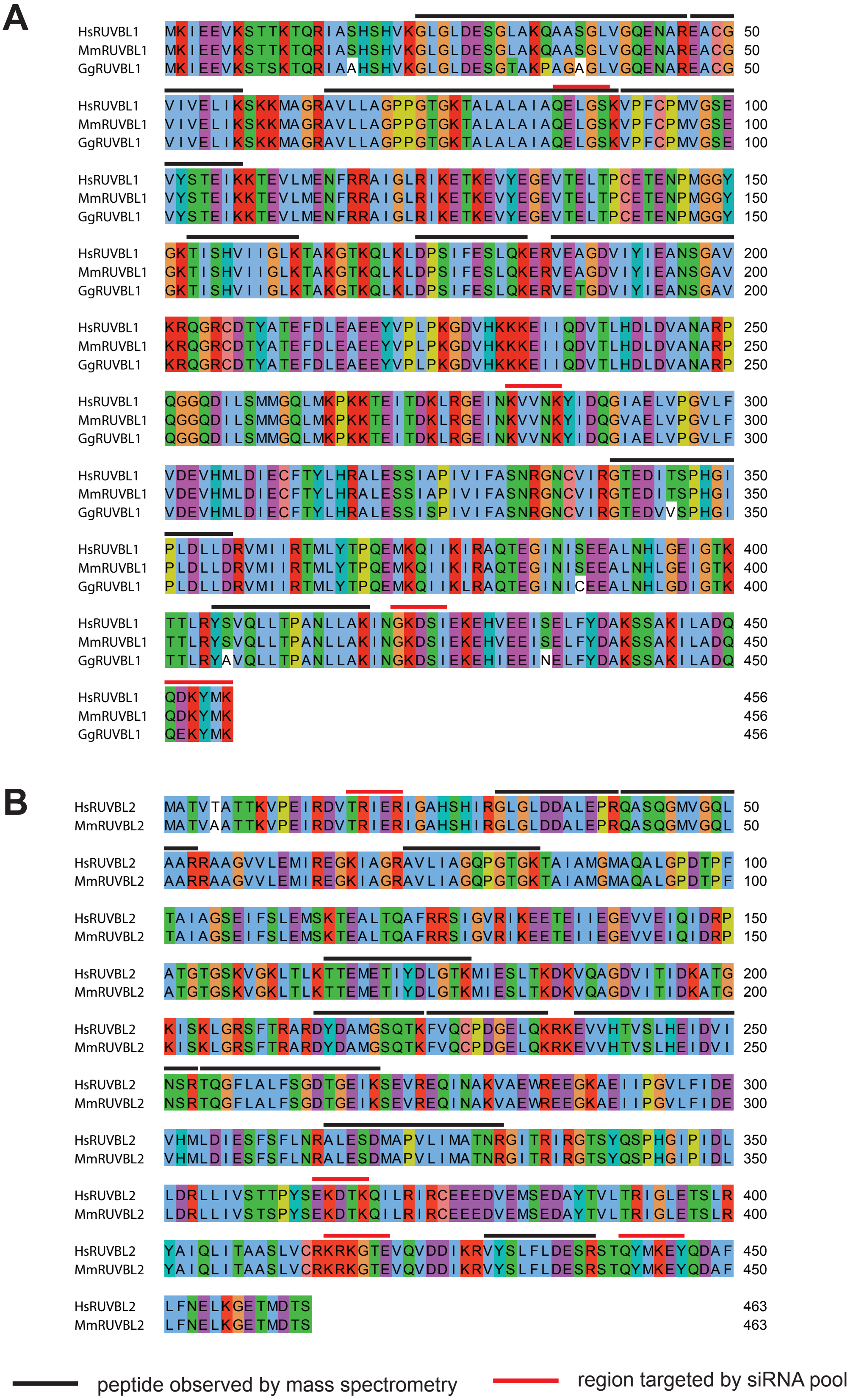

Figure S1. RuvBL1 and RuvBL2 are highly conserved. Protein sequence alignments generated with ClustalW demonstrate strong conservation of vertebrate RuvBL1 (**A**) and human and mouse RuvBL2 (**B**). *Gallus gallus* RuvBL2 has not been detected in the published chicken proteome owing to incomplete annotation. Red bars indicate regions targeted with siRNAs in the Dharmacon ON-TARGET SMARTpool. Black bars lines indicate peptide regions identified by mass spectrometry of the purified FA core complex. In the case of RuvBL2 mass spectrometry, peptides from the *Bos taurus* and *Xenopus laevis* orthologues were detected. The alignment has been coloured by ClustalX notation. Each residue is coloured based on the occurrence of particular residues at the specified position: Blue (A, I, L, M, F, W, V), Red (R, K), Green (N, Q, S, T), Pink (C), Magenta (E, D), Orange (G), Cyan (H, Y) and Yellow (P).

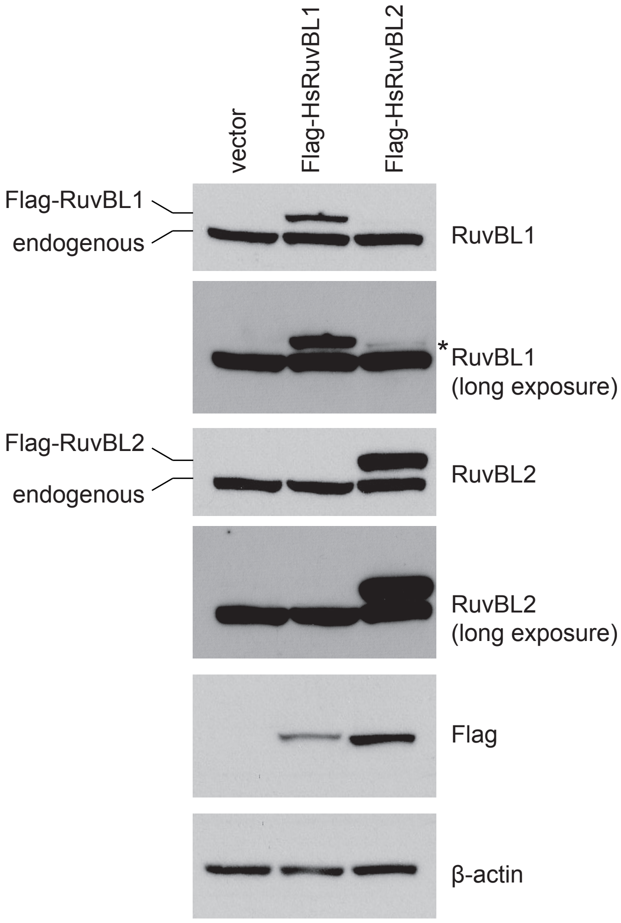

Figure S2. Antibodies against RuvBL1 and RuvBL2 are specific. FLAG-RuvBL1 or FLAG-RuvBL2 was overexpressed in U2OS cells. WCEs were blotted with antibodies against both proteins. The antibody against RuvBL1 shows mild cross-reactivity with the overexpressed FLAG-RuvBL2 (*). The anti-RuvBL2 antibody did not cross-react with FLAG-RuvBL1.

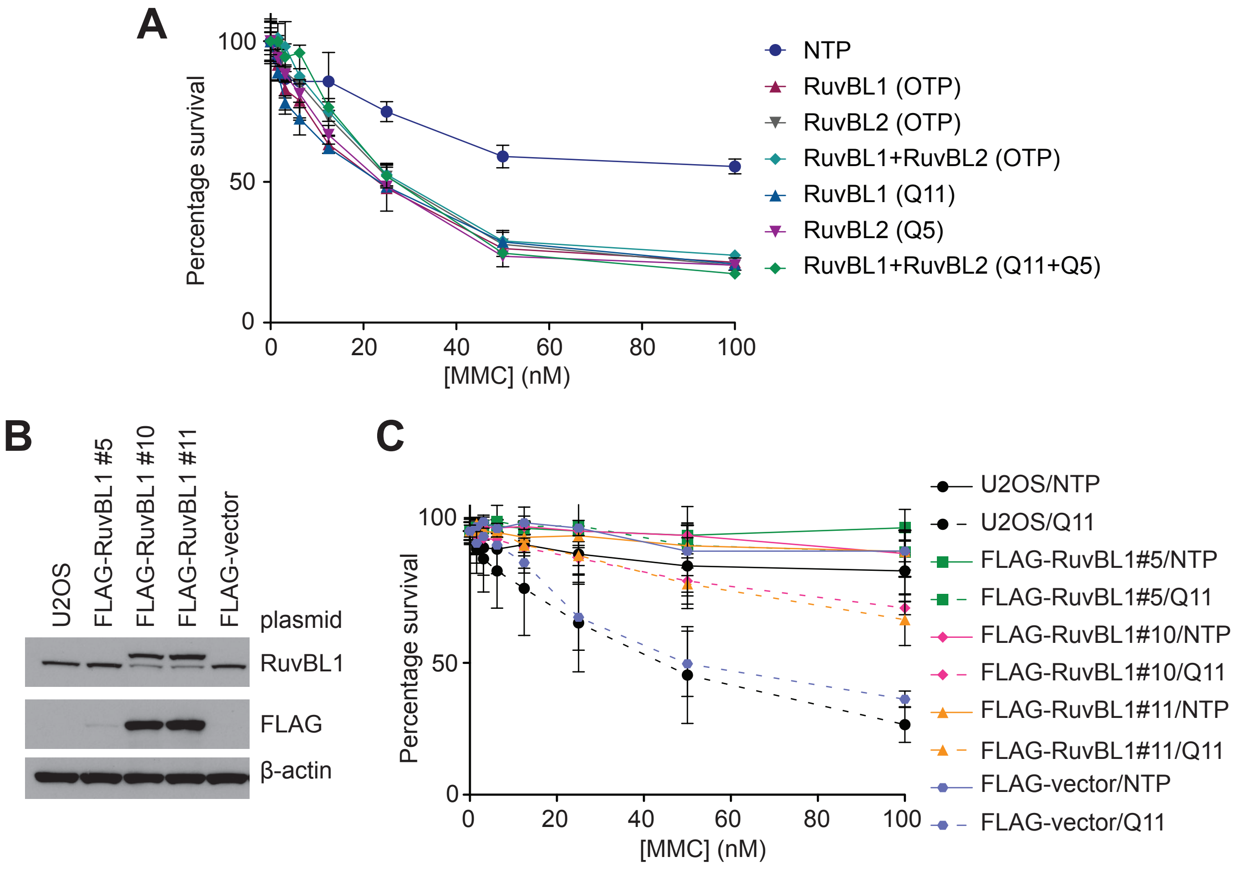

Figure S3. siRNA-mediated depletion of RuvBL1-RuvBL2 in human cells confers sensitivity to DNA crosslinking agents. (**A**) siRNA-mediated depletion of RuvBL1 and/or RuvBL2 using either a Dharmacon OTP SMARTpool of four distinct siRNAs targeting the coding region (OTP) or single siRNAs targeting the 3'-UTR (Q11, Q5) confers similar cellular hypersensitivity to MMC. These results are in agreement with the experiments in Figure 2B. Each point represents the mean of triplicate samples and the error bars are the SD. (**B,C**) U2OS cells were stably transfected with empty vector or plasmid encoding siRNA-resistant FLAG-RuvBL1. WCEs from three independent FLAG-RuvBL1 clones, vector control and untransfected cells (U2OS) were analysed by Western blotting with indicated antibodies (**B**). Stable overexpression of FLAG-RuvBL1 caused partial down-regulation of the endogenous RuvBL1 protein. It was not possible to obtain cell lines stably expressing a catalytic-dead mutant of RuvBL1 (D302N) suggesting that this protein may function in a dominant negative manner. Stable cell lines expressing siRNA-insensitive FLAG-RuvBL1 rescue MMC sensitivity after depletion of endogenous RuvBL1 (**C**). MMC sensitivity was determined after treatment with a non-targeting pool of siRNA (NTP; solid lines) or with a siRNA targeting the 3'-UTR of endogenous RuvBL1 not encoded in the overexpression construct (Q11; dotted lines). Both U2OS cells (black) and a cell line with a stably integrated empty vector (blue) were hypersensitive to MMC after RuvBL1 knockdown. However, three independent clones expressing FLAG-RuvBL1 (green, pink, orange) showed partial to complete rescue of sensitivity. Each point represents the mean of triplicate samples and the error bars are the SD.

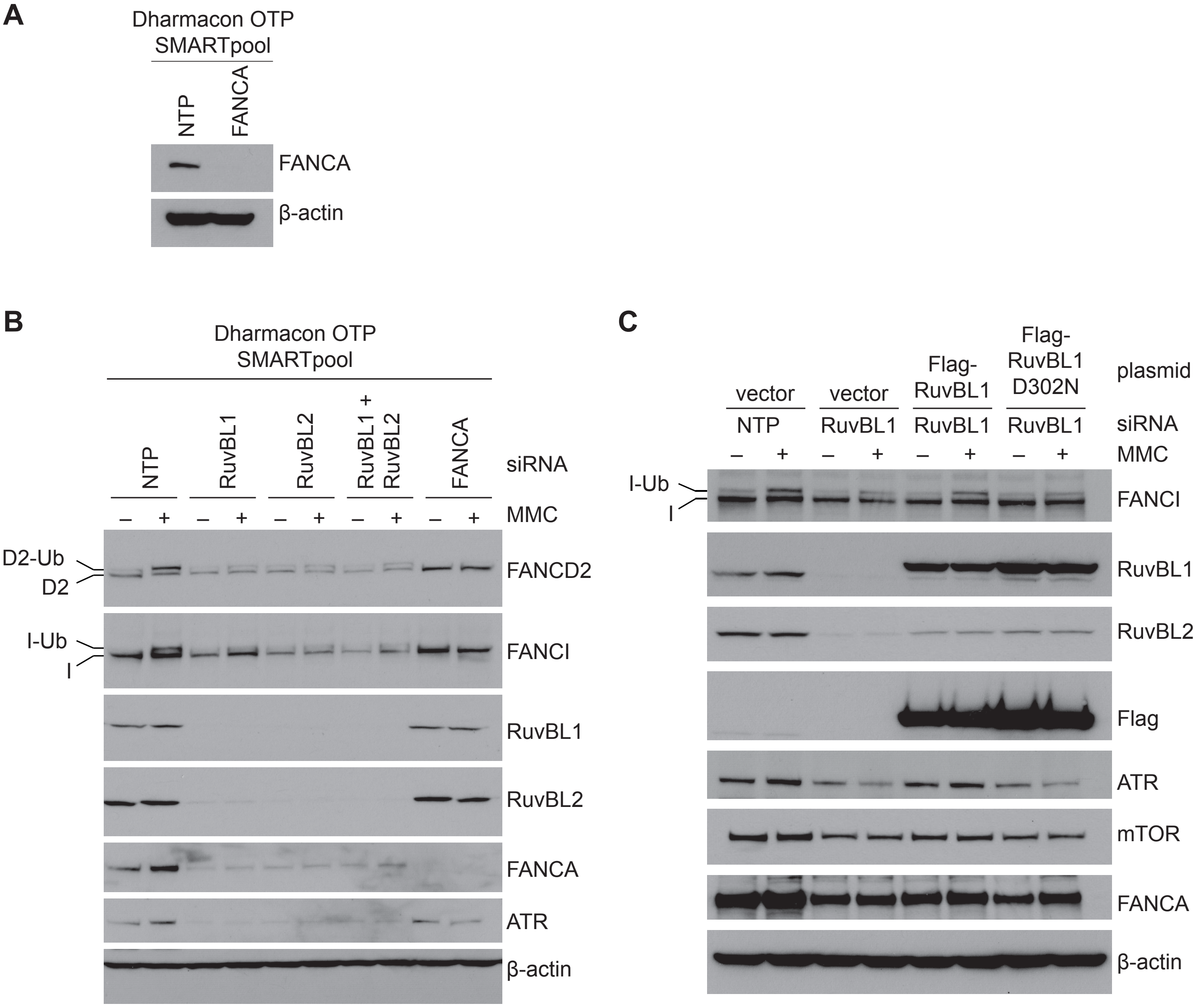

Figure S4. RuvBL1-RuvBL2 depletion impairs the activity and abundance of the FA core complex which can be rescued with siRNA-insensitive RuvBL1. (**A**) A Dharmacon OTP SMARTpool siRNA set targeting human FANCA specifically depleted the FANCA protein as detected by Western blotting of WCEs in U2OS cells using a specific FANCA antibody. NTP is a non-targeting pool control siRNA. (**B**) Knockdown of RuvBL1 and RuvBL2 using Dharmacon OTP SMARTpool siRNAs causes a defect in DNA damage-induced monoubiquitination of FANCD2, a severe reduction in FANCI monoubiquitination and reduced ATR and FANCA protein levels in U2OS cells. These results are in agreement with the same experiments in Figure 3B performed with single siRNAs. Cells were treated with indicated siRNAs and exposed to MMC or left untreated. WCEs were blotted with the indicated antibodies. (**C**) FA defects after RuvBL1 depletion are rescued by wild-type but not ATPase deficient RuvBL1: U2OS WCEs were blotted with indicated antibodies after simultaneous depletion of RuvBL1 with a siRNA targeting the 3'-UTR (Q11) and transient overexpression of a cDNA encoding FLAG-RuvBL1 (insensitive to siRNA) or a vector control. FLAG-RuvBL1 expression rescued protein levels of ATR, mTOR, RuvBL2 and FANCA, and monoubiquitination of FANCI. Overexpression of an empty vector or catalytic-dead mutant (D302N) were not able to functionally rescue RuvBL1 depletion. The D302N mutant partially rescued the level of RuvBL2 indicating that the catalytic mutant likely retains the ability to form a higher order, albeit inactive, heteromeric assembly with RuvBL2.

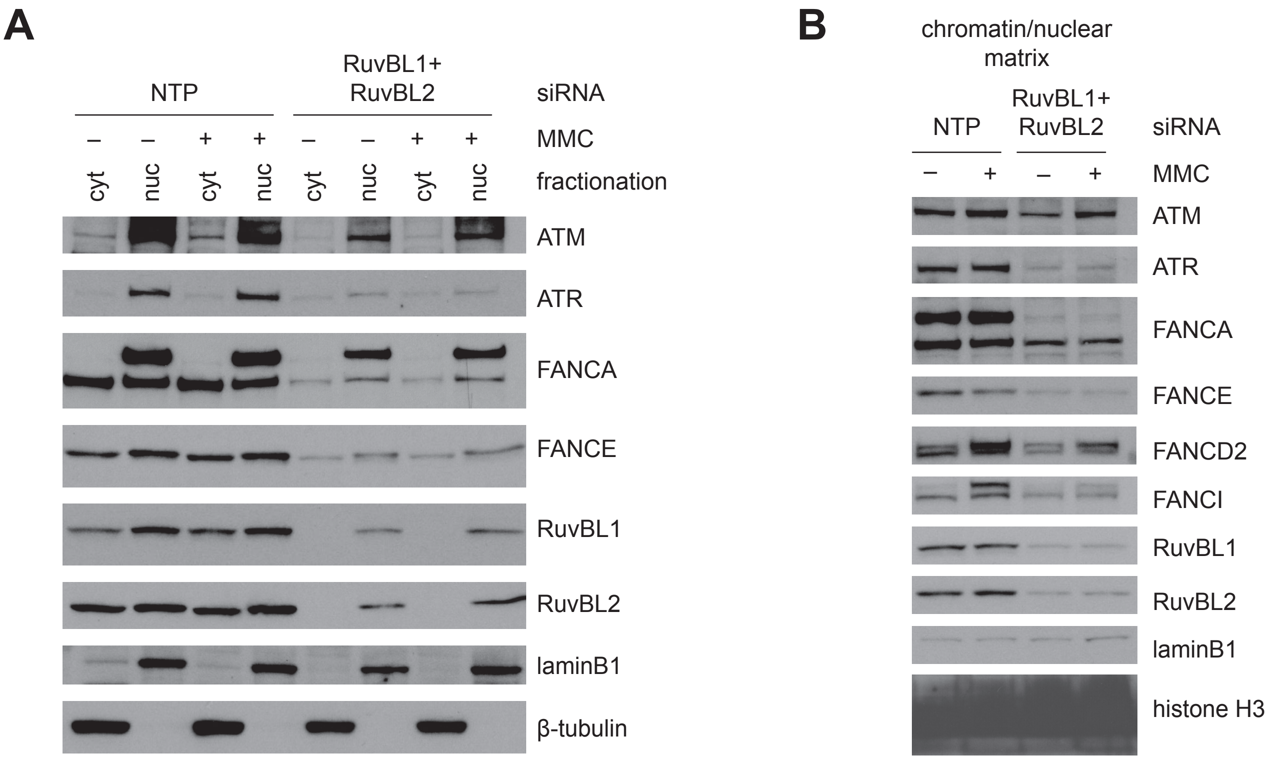

Figure S5. Subcellular localisation of the Fanconi Anaemia core complex is not impaired in RuvBL1-RuvBL2-depleted cells. U2OS cells were treated with either a non-targeting siRNA pool (NTP) or single siRNAs targeting both RuvBL1 and RuvBL2 and exposed to 1 μM MMC or left untreated. Cells were fractionated into (**A**) cytoplasmic (cyt) and nuclear (nuc) compartments or (**B**) a chromatin-enriched fraction and subjected to blotting with indicated antibodies. Although the abundance of the FA core complex is reduced after RuvBL1-RuvBL2 depletion, it is localised normally relative to cells treated with the control siRNA. Weak monoubiquitination of FANCI is clearly observed on chromatin, where the monoubiquitinated species are heavily enriched *in vivo*, suggesting that RuvBL1-RuvBL2 depletion severely reduces, but does not fully abrogate, this modification. The presence of residual RuvBL1 and RuvBL2 in the same fraction, likely a consequence of incomplete siRNA-mediated suppression, could also account for residual monoubiquitinated substrates. β-tubulin serves as a cytoplasmic loading control; laminB1 serves as a nuclear/nuclear matrix loading control; and histone H3 serves as a chromatin loading control. It is not clear whether the upper band in the FANCA panel is a modified form of FANCA or a cross-reacting protein.

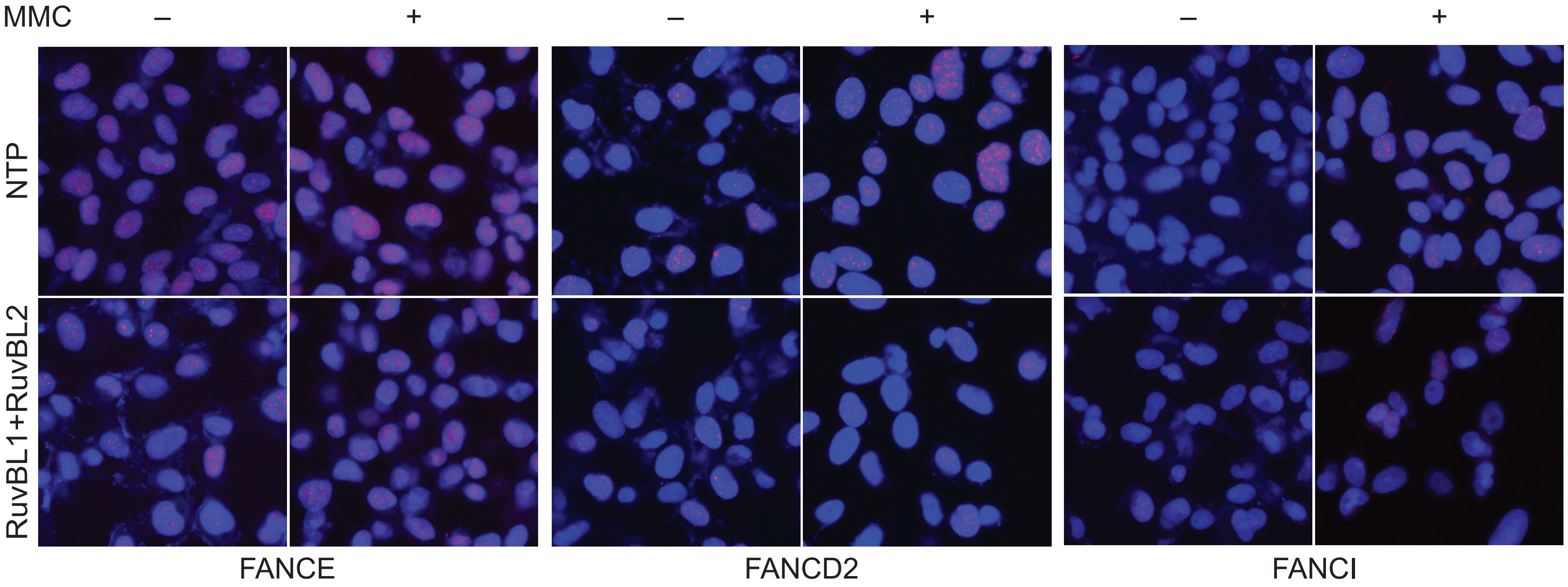

Figure S6. FANCE, FANCD2 and FANCI are targeted to subnuclear foci after RuvBL1-RuvBL2 depletion. Indirect immunofluorescence using a FANCE, FANCD2 or FANCI antibody in U2OS cells treated with either a non-targeting siRNA pool (NTP) or siRNAs targeting both RuvBL1 and RuvBL2. The formation of nuclear FANCE foci (which occur independent of MMC-induced damage) is intact. Although FANCD2 and FANCI are not efficiently monoubiquitinated after RuvBL1-RuvBL2 depletion, they can still localise to chromatin and are able to form nuclear foci, albeit in a reduced capacity. DAPI staining of nuclei is in blue and FANCE, FANCD2 and FANCI are in magenta.

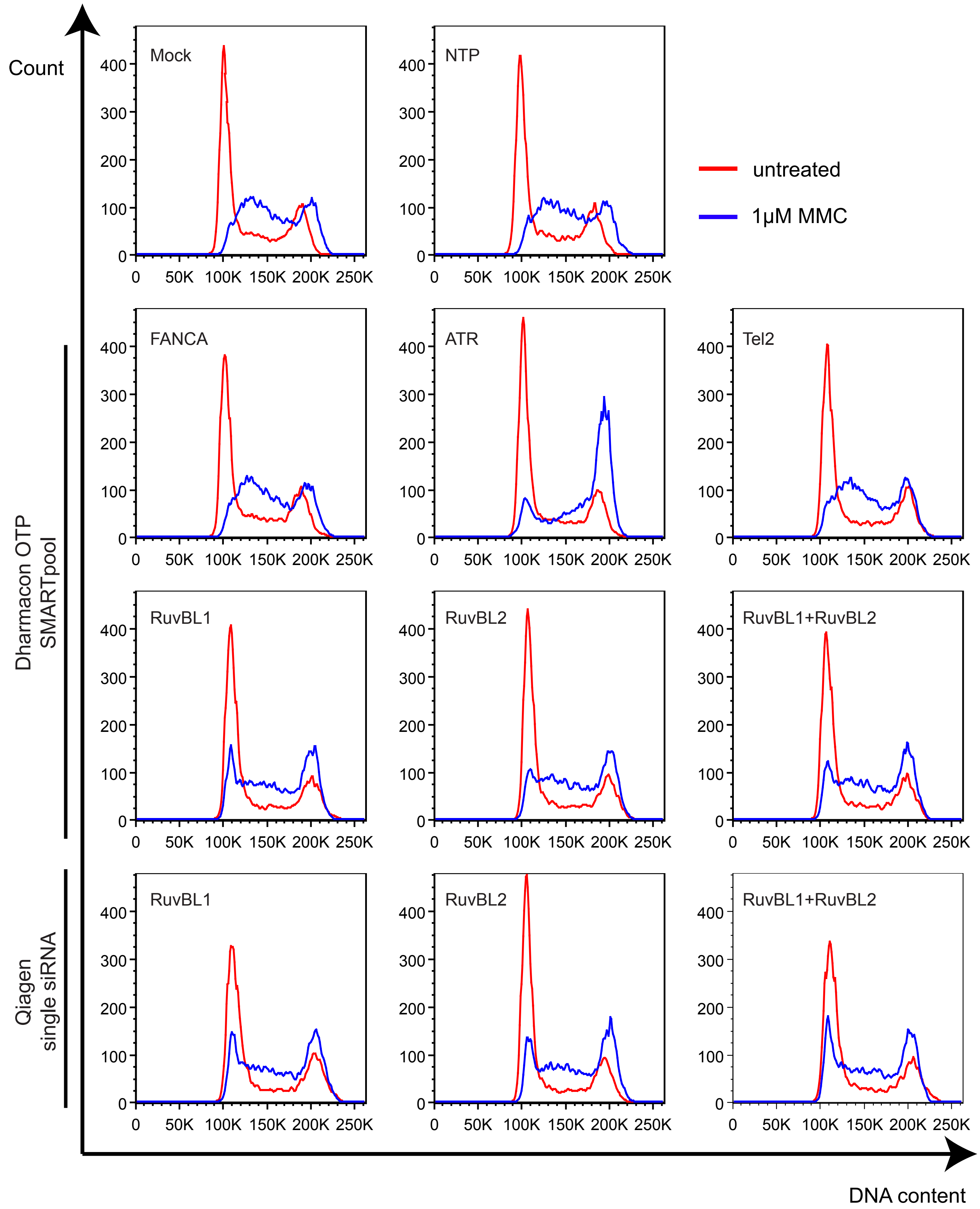

Figure S7. Cell cycle analysis of RuvBL1-RuvBL2-depleted cells before and after MMC treatment. Representative flow cytometric profiles of propidium iodide (PI)-stained asynchronous U2OS cells after the indicated siRNA treatment either in the absence (red) or presence (blue) of 1 µM MMC. 10 000 events were counted per sample. The cell cycle profiles of RuvBL1 and RuvBL2 knockdown together, or in isolation, are very similar, providing further evidence of their functional epistasis. In cells not treated with MMC, the flow cytometric profiles of RuvBL1-RuvBL2 depleted cells match the other siRNA treatments (FANCA, ATR, Tel2). MMC-induced DNA damage causes a G2-arrest. A mild defect in replication onset is marked by an increased G1 population. Overall, cell cycle profiles show that cells do not substantially accumulate at a specific cell cycle stage and therefore this is compatible with Fanconi Anaemia pathway activation. NTP, non-targeting siRNA pool.

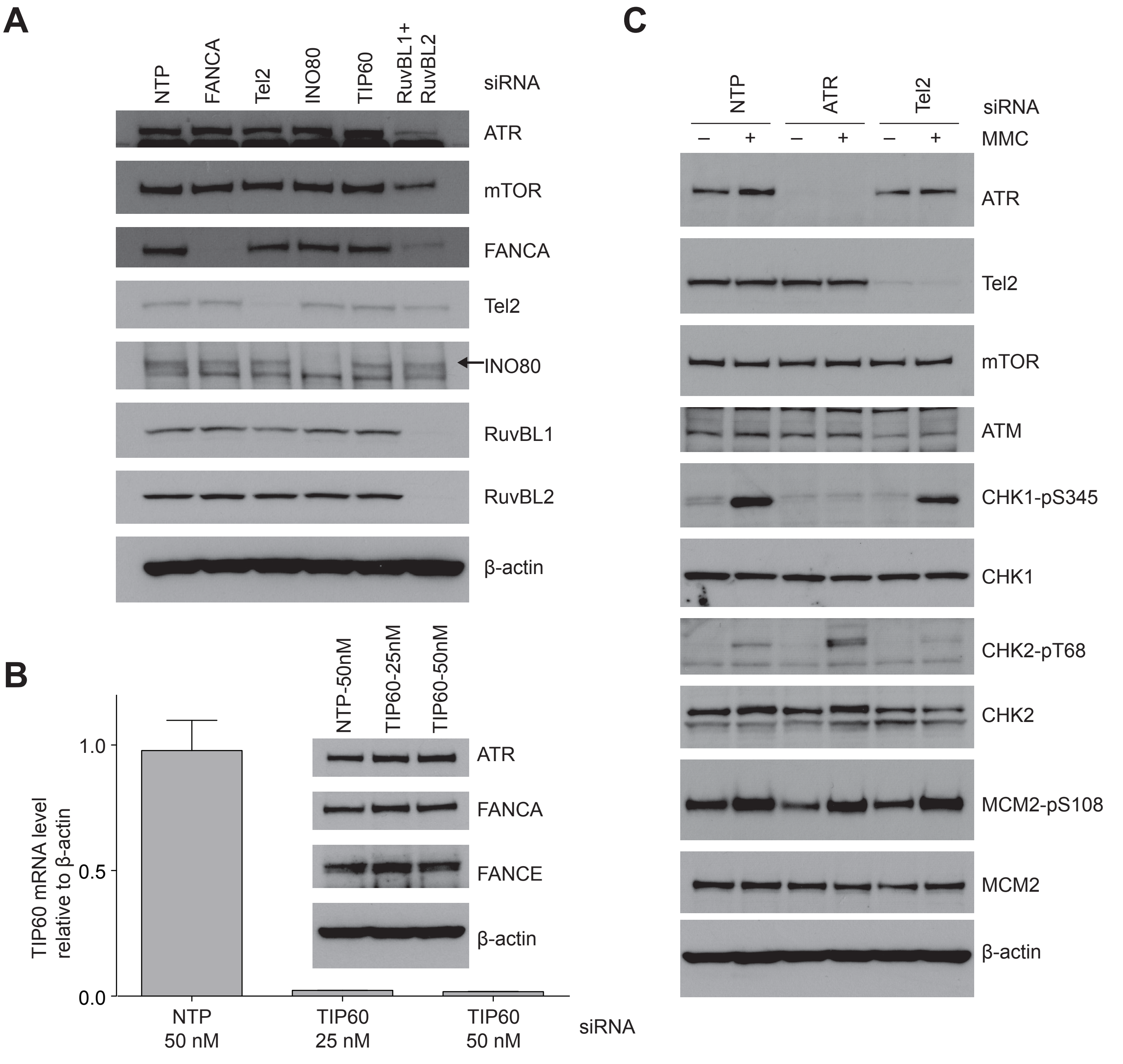

Figure S8. Regulation of the abundance of the FA core complex by RuvBL1-RuvBL2 occurs independently of the INO80 and TIP60 complexes. (**A**) U2OS cells were treated with the indicated siRNAs targeting the FA core complex (FANCA), the PIKK chaperone Tel2, the INO80 chromatin remodeler complex (INOC1), the TIP60 histone acetyltransferase complex (TIP60) and RuvBL1-RuvBL2. WCEs were blotted with indicated antibodies. Only depletion of FANCA or RuvBL1-RuvBL2 caused reduction of FANCA protein levels. TIP60 could not be reliably detected by Western blotting. (**B**) siRNA-mediated depletion of TIP60 was additionally performed at a higher siRNA dose (50 nM) and WCEs from treated U2OS cells were analysed by Western blotting. No depletion of FANCA or FANCE was observed (inset). To confirm suppression of TIP60, real-time quantitative PCR was used to determine the mRNA level of TIP60 after knockdown. TIP60 siRNA caused significant reduction of TIP60 mRNA at the indicated doses (bar chart). (**C**) To confirm the functional efficiency of their suppression, known phenotypes associated with siRNA-mediated depletion of ATR and Tel2 were analysed by Western blotting of U2OS WCEs in the absence and presence of MMC treatment. ATR depletion caused impairment of CHK1, but not CHK2, activation and a reduction in MCM2 phosphorylation. Tel2 depletion caused a mild reduction in the abundance of PIKKs ATM, ATR and mTOR.

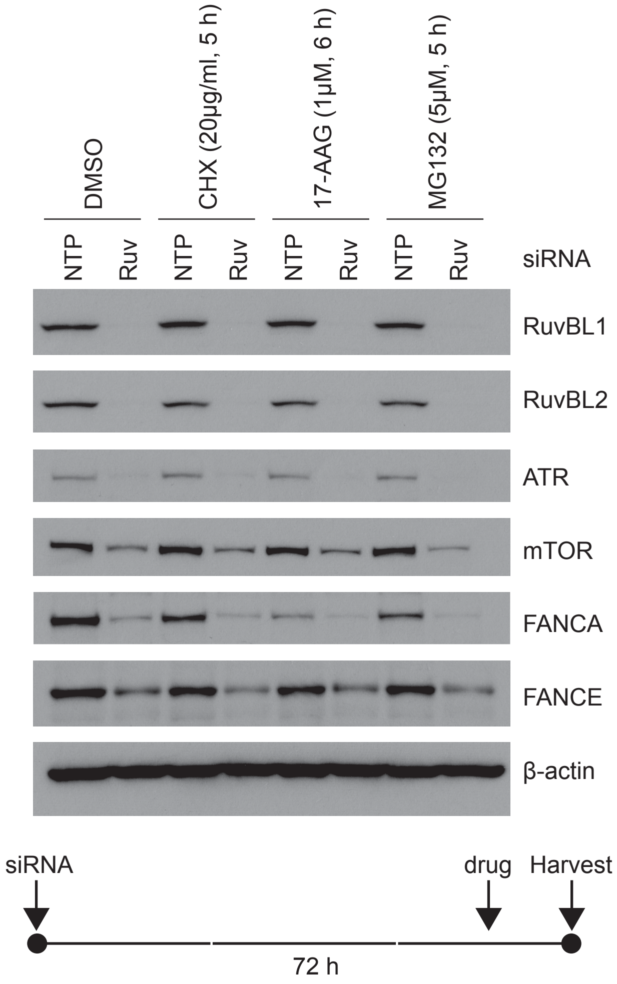

Figure S9. Regulation of the abundance of the FA core complex by RuvBL1-RuvBL2 is not influenced by translational inhibition, HSP90 inhibition or proteasome-mediated degradation. U2OS cells were treated with either a non-targeting siRNA pool (NTP) or single siRNAs targeting both RuvBL1 and RuvBL2 (Ruv) and exposed to the indicated doses of translation inhibitor cyclohexamide (CHX), HSP90 inhibitor 17-AAG or proteasome inhibitor MG132 for indicated times. WCEs were analysed by Western blotting with indicated antibodies. Significant changes in FANCA and FANCE protein levels were not observed in control samples after CHX treatment. HSP90 inhibition specifically caused depletion of FANCA, as previously reported (90) but not FANCE. The effect of 17-AAG on FANCA abundance appears additive and mechanistically distinct from RuvBL1-RuvBL2-mediated reduction. MG132 is able to prevent 17-AAG-induced degradation of FANCA (90) but critically does not inhibit RuvBL1-RuvBL2-mediated loss of FANCA and FANCE. FANCA and FANCE levels could not be rescued by proteasomal inhibition with MG132. These effects were also observed with the PIKKs ATR and mTOR whose abundance is also regulated by RuvBL1-RuvBL2 (31).

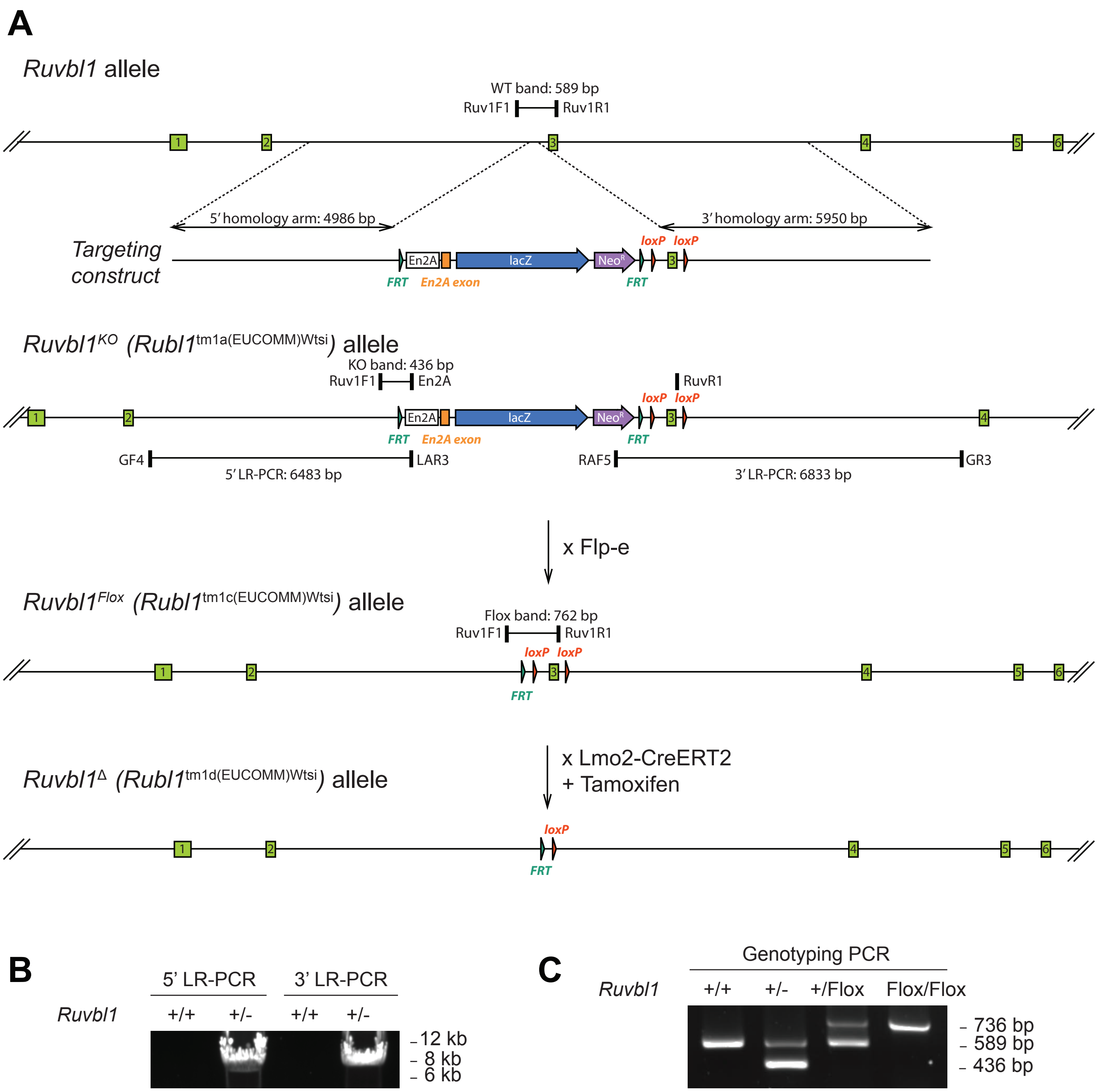

Figure S10. Generation of *Ruvbl1* conditional knockout mice. (**A**) Map of the *Ruvbl1* locus and the targeting construct used to electroporate embryonic stem cells. The *Ruvbl1*$^{\text{tm1a(EUCOMM)Wtsi}}$ synthetic allele generated, following homologous recombination, is predicted to disrupt *Ruvbl1* leading to no protein expression. Mice carrying the *Ruvbl1*$^{\text{tm1a(EUCOMM)Wtsi}}$ allele were crossed with mice carrying a Flp-e recombinase transgene, resulting in recombination between *FRT* sites and the generation of the *Ruvbl1*$^{\text{tm1c(EUCOMM)Wtsi}}$ or *Ruvbl1*^{Flox} allele, which is predicted to restore protein expression. Finally, *Ruvbl1*$^{\text{Flox/Flox}}$ mice were crossed with mice carrying the CreERT2 recombinase under the control of the Lmo2 promoter which, upon tamoxifen treatment, should lead to deletion of the third exon in the *Ruvbl1* locus (*Ruvbl1*$^{\text{tm1d(EUCOMM)Wtsi}}$ allele) in haematopoietic stem cells.
(**B**) Targeted integration of the targeting construct by homologous recombination was confirmed by long range PCR. Oligonucleotide pairs were used in which one oligo binds within the targeting construct whilst the other binds externally to the homology arms. Targeting of the 5' arm was confirmed using oligos GF4 and LAR3, whilst the 3' arm was confirmed by RAF5 and GR3. Both PCR reactions generated products of the predicted size of 6483 bp and 6833 bp, respectively.
(**C**) Following blastocyst injection of the targeted ES cells, germline transmission was achieved. The progeny were genotyped using a PCR strategy in which the wild type allele was amplified with oligos Ruv1F1 and Ruv1R1, generating a 589 bp product. The targeted allele *Ruvbl1*$^{\text{tm1a(EUCOMM)Wtsi}}$ was amplified with oligos Ruv1F1 and En2A generating a 436 bp product. The same PCR strategy was able to detect recombination between *FRT* sites and to genotype mice carrying the *Ruvbl1*^{Flox} allele (736 bp).

Supplementary Table 1: siRNAs used in this study

Target	Supplier	Sequence
RuvBL1	Dharmacon	AUAAGGUGGUGAACAAGUA
		GGGAAGGACAGCAUUGAGA
		CAGGAUAAGUACAUGAAGU
		CUCAGGAGCUGGGUAGUAA
RuvBL2	Dharmacon	UAACAAGGAUUGAGCGAAU
		CGCAGUACAUGAAGGAGUA
		GAAACGCAAGGGUACAGAA
		GCGAGAAAGACACGAAGCA
FANCA	Dharmacon	GGGCCAUGCUUUCUGAUUU
		GCAGGUCACGGUUGAUGUA
		GUAGAAGGUCCACUGUGUA
		GUUAGAGUUUGCUCAGUAU
ATR	Dharmacon	GAGAAAGGAUUGUAGACUA
		GCAACUCGCCUAACAGAUA
		CCACGAAUGUUAACUCUAU
		CCGCUAAUCUUCUAACAUU
Tel2	Dharmacon	UGAUGUGCCUGGCUGUUAA
		CCCUGAAAUUCCAGUACGA
		UGGAGUCCCUGAAGCGGUA
		UGGCCAGAUUCCUGCGCGA
Non-targeting	Dharmacon	
RuvBL1 (Q10)	Qiagen (rescue)	TCTATAAATCTTCATAGGTTA
RuvBL1 (Q11)	Qiagen (rescue)	CCTGTGGTTGCTTTGAAAGAA
RuvBL2 (Q5)	Qiagen (rescue)	CTGACACTGTGACTCTGTATA
RuvBL2 (Q6)	Qiagen (rescue)	TTTCCACCAGAGTTCTGACAC
INOC1	Dharmacon	GGAAUUGAGUUUCGAUAGA
		GGAGUUAUUUGAACGGCAA
		GAAUCAACUUUCUCGCUUA
		GAGGAAACCAACCGAGUGA
HTATIP (TIP60)	Dharmacon	CGUAAGAACAAGAGUUAUU
		AUGAAUGGGUGACGCAUGA
		GGACAGCUCUGAUGGAAUA
		GACCAAGUGUGACCUACGA

Dharmacon = ON-TARGETplus SMARTpool comprising four siRNAs targeting the coding region of the gene

Qiagen = rescue siRNA, a single siRNA targeting the 3'-UTR of the gene

Supplementary Table 2: Quantitect Primer Assays used in this study (RT-PCR)

Target gene	Assay name
β-actin	Hs_ACTB_2_SG
FANCA	Hs_FANCA_1_SG
FANCB	Hs_FANCB_2_SG
FANCL	Hs_FANCL_1_SG
FANCM	Hs_FANCM_2_SG
TIP60	Hs_KAT5_1_SG

Supplementary Table 3: Antibodies used in this study

Target	Supplier	Code	Dilution	Species
ATM (2C1)	Abcam	AB78	1:2500	Human
ATR (N-19)	Santa Cruz	1877	1:2500	Human
β-actin (AC-15)	Sigma	A5441	1:10000	Human
CHK1 (G-4)	Santa Cruz	8408	1:2000	Human
CHK1-S317	Cell Signaling Technology	2344	1:1500	Human
CHK1-S345	Cell Signaling Technology	2341	1:1500	Human
CHK2	ProSci Inc	2391	1:2000	Human
CHK2-T68	Cell Signaling Technology	2661	1:1500	Human
DNA-PKcs (C-19)	Santa Cruz	1552	1:1000	Human
FANCA	Fanconi Anemia Research Fund	R6512	1:1000	Human
FANCD2	non-commercial (43)		1:3000	Chicken
FANCD2	non-commercial (59)		1:3000	Human
FANCE	non-commercial (59)		1:1500	Human
FANCI	Abcam	AB15344	1:3000	Human
Flag (M2)	Sigma	F1804	1:5000	Human
Histone H3	Abcam	AB1791	1:40000	Human
INO80	Abcam	AB118787	1:3000	Human
LaminB1 (M-20)	Santa Cruz	6217	1:2000	Human
MCM2	Abgent	AB9377b	1:2000	Human
MCM2-S108P	Bethyl Laboratories	A300-094A	1:8000	Human
mTOR	Cell Signaling Technology	7C10	1:2500	Human
RuvBL1 (2943C1a)	Abcam	AB51500	1:1000	Human/Chicken
RuvBL2	BD	612482	1:1000	Human/Chicken
Tel2	Proteintech Group	15975-1-AP	1:1000	Human
β-tubulin (2.1)	Sigma	T4026	1:20000	Human

Supplementary Materials and Methods

Cellular fractionation. For biochemical fractionation of U2OS cells into cytoplasmic/nuclear compartments, cells were fractionated using the Subcellular Protein Fractionation Kit (Thermo Scientific) according the manufacturer's instructions. For chromatin/nuclear matrix isolation, the protocol described in (91) was performed with minor modifications as described henceforth. U2OS cells were harvested by trypsinisation, washed in PBS and resuspended in 150 µl buffer A (10 mM HEPES, pH 7.9, 10 mM KCl, 1.5 mM $MgCl_2$, 0.34 M sucrose, 10% glycerol, 1 mM DTT, 1 mM PMSF, 1X Protease Inhibitor Cocktail, 2 mM Na_3VO_4, 10 mM NaF, 1 µM Okadaic acid). Triton X-100 was added to a final concentration of 0.05%. Nuclei (P1) were collected by low speed centrifugation (1300 x g, 4 min, 4°C). The nuclear pellet was washed in buffer A, to remove cell debris and insoluble aggregates, and lysed in 120 µl buffer B (3 mM EDTA, 0.2 mM EGTA, 1 mM DTT, 1 mM PMSF, 1X protease inhibitors, 2 mM Na_3VO_4, 10 mM NaF, 1 µM Okadaic acid). Centrifugation of P1 (1700 x g, 4 min, 4°C) pelleted insoluble chromatin (P3) from the soluble nuclear fraction. The P3 chromatin pellet was resuspended in buffer A adjusted with 1 mM $CaCl_2$ and 6 U micrococcal nuclease and incubated for 10 min at 37°C. The reaction was quenched by addition of EGTA to a final concentration of 1 mM. High-speed centrifugation (20 000 x g, 10 min, 4°C) was used to collect the supernatant containing the chromatin fraction, P3. Proteins were quantitated using the BCA assay and made up in 4X LDS sample loading buffer (Invitrogen). 20 µg P3 was loaded for each treatment and analysed by SDS-PAGE followed by Western blotting.

Flow cytometry. U2OS cells were incubated for 48 h with indicated siRNAs. 1 µM MMC or vehicle control was added for a further 24 h. After siRNA and/or drug treatment, U2OS cells were harvested by trypsinisation, washed twice in PBS, fixed in ice-cold 70% ethanol and incubated for 72 h at -20°C. Cells were again washed in ice-cold PBS and stained in propidium iodide solution to measure DNA content. Samples were analysed by flow cytometry on a Becton Dickinson LSRII Flow Cytometer using standard gatings and counting 10 000 events/sample. FACS profiles were analysed using FloJo 9.3.2.

Immunofluorescence. U2OS cells were treated with siRNA and/or MMC and grown on glass coverslips in 6 well plates. For FANCE detection, immunofluoresence was performed essentially as previously described (59). Briefly, cells were washed twice in PBS, fixed in ice-cold methanol and incubated at -20°C for 10 minutes. After three further PBS washes, cells were permeabilised in 0.5% Igepal CA-630/PBS for 10 min and washed again in PBS. Samples were blocked for 1 h in blocking buffer (1% BSA, 0.05% Triton X-100, PBS) and incubated overnight in a humidified chamber with primary antibody at 1/1000 at 4°C. The following day, cells were washed three times in blocking buffer and incubated for 1 h with Alexa Fluor 568-conjugated goat anti-rabbit IgG secondary antibody (Invitrogen) in the dark. Cells were washed three times in blocking buffer and twice in PBS before mounting in Vectashield Mounting Media with DAPI (Vector Laboratories). For FANCD2 and FANCI

detection, cells were treated as above except they were pre-extracted with 0.5% Triton X-100 in PBS prior to fixation in 4% formaldehyde/PBS. Cells were imaged using a Zeiss LSM710 inverted confocal microscope and imported into ImageJ software (http://rsbweb.nih.gov/ij/) using the full range of pixel intensities.

Mammalian transfection. For siRNA treatment, U2OS cells were reverse transfected with 25 nM siRNA (Dharmacon and Qiagen; Table S1) using Dharmafect 1 reagent (Dharmacon) according to the manufacturer's protocol. Unless stated otherwise, cells were incubated for 48 h with indicated siRNAs. 1 µM MMC, or vehicle control, were added for a further 24 h. For Western blotting and immunofluoresence, 2.5 x 10^5 cells/well of a 6-well plate were transfected. For metaphase spreads and subcellular fractionation, 1.2 x 10^6 cells were transfected in a 10 cm dish. For co-transfection, cells were transfected in a 6-well plate with siRNA and 450 ng plasmid, with JetPRIME reagent according the manufacturer's protocol. For stable transfection, U2OS cells were transfected in a 10 cm dish with 10 µg plasmid with JetPRIME reagent. After 24 h, cells were changed into fresh media and after a further 24 h, media was supplemented with G418 at a final concentration of 0.5 mg/ml. Cells were left under selection until stable clones developed and isolated colonies were picked, expanded and correct expression verified by Western blotting.

Metaphase spreads. After siRNA treatment and/or drug treatment, U2OS cells were treated with 100 ng/ml colcemid 4 h prior to harvest. Cells were collected by mitotic shake-off and pooled with those collected by trypsinisation to enrich for mitotic cells. Cells were gently hypertonically swollen in 75 mM KCl at room temperature before fixation in freshly prepared Carnoy's solution (methanol: acetic acid, 3:1). Fixed cells were dropped onto ethanol-rinsed microscope slides and dried at 50°C for 20 min. Slides were stained with Giemsa diluted in pH 6.8 Gurr buffer and mounted with Eukitt Mounting Media (Sigma). Metaphases were imaged using an Olympus BX60 microscope and scored blind to the observer.

Plasmid construction. FLAG-RuvBL1 and FLAG-RuvBL2 were subcloned from human I.M.A.G.E. cDNA clones (ID: 100004078 and ID: 100009028, respectively, from Source BioScience LifeSciences) using PCR with *Not*I and *Kpn*I adapted primers into *Not*I and *Kpn*I sites in p3XFLAG-CMV-10 (Sigma). Constructs were verified by DNA sequencing. The D302N mutation in FLAG-RuvBL1 was introduced using the primer pair 5'-gtgctgtttg ttaatgaggt ccacatgc and 5'-gcatgtggac ctcattaaca aacagcac and the QuikChange II XL Site-Directed Mutagenesis Kit (Agilent Technologies) according to the manufacturer's instructions.

Sequence alignments. Sequence alignments were performed with ClustalW and further processed and rendered with JalView 2.7 (92).

Supplementary References

90. Oda, T., Hayano, T., Miyaso, H., Takahashi, N. and Yamashita, T. (2007) Hsp90 regulates the Fanconi anemia DNA damage response pathway. *Blood*, **109**, 5016–5026.

91. Méndez, J. and Stillman, B. (2000) Chromatin association of human origin recognition complex, cdc6, and minichromosome maintenance proteins during the cell cycle: assembly of prereplication complexes in late mitosis. *Molecular and Cellular Biology*, **20**, 8602–8612.

92. Clamp, M., Cuff, J., Searle, S.M. and Barton, G.J. (2004) The Jalview Java alignment editor. *Bioinformatics*, **20**, 426–427.
